# Supplementary material for: Identification of photoperiod-induced specific miRNAs in the adrenal glands of Sunite sheep (Ovis aries)
Source: Front Vet Sci. 2022 Jul 22;9:888207. doi: 10.3389/fvets.2022.888207 (PMC9354845; doi:10.3389/fvets.2022.888207)
Supplement: Supplementary file 1 [file Data_Sheet_1.ZIP › Supplementary file/miRDeep2 file.zip!/miRDeep2 file/Know_Structure/SPLP42A1/oar-mir-150.pdf]

miRBase precursor	: oar-mir-150
Total read count	: 266
oar-miR-150 read count	: 266
remaining reads	: 0

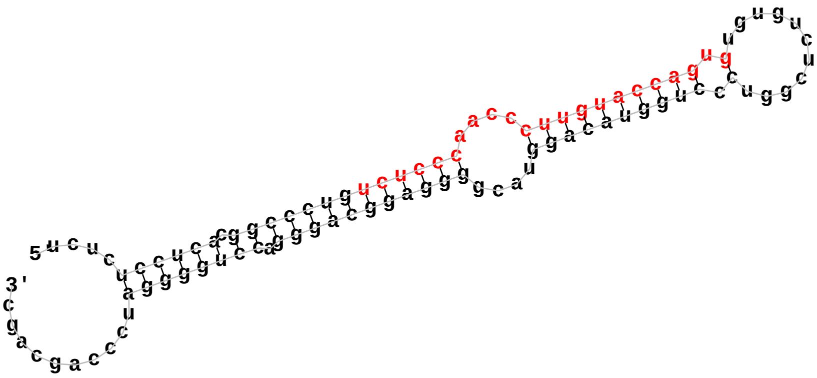

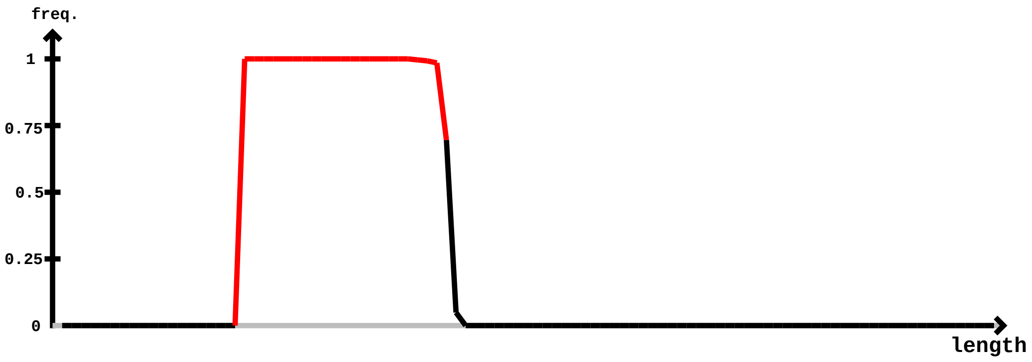


```
                  oar-miR-150
5'- ucucuccucacggcccugucucccaacccuuguaccagugugugucucggucccugguacagguacggggaggcagggaccuggggaucccagcagc   -3'    exp
    ....((((((.(((((((((((((....((((((((((.(...........).))))))))))....))))))))))).)))))))))..........   reads    mm    sample
    ..................ucucccaacccuuguacc...............................................................   1        0     seq
    ..................ucucccaacccuuguacca..............................................................   1        0     seq
    ..................ucucccaacccuuguaccag.............................................................   2        0     seq
    ..................ucucccaacccuuguaccagu............................................................   77       0     seq
    ..................ucucccaacccuuguaccagug...........................................................   172      0     seq
    ..................ucucccaacccuuguaccagugu..........................................................   13       0     seq
```
